# Predicting Persistent Back Symptoms by Psychosocial Risk Factors: Validity Criteria for the ÖMPSQ and the HKF-R 10 in Germany

**DOI:** 10.1371/journal.pone.0158850

**Published:** 2016-07-21

**Authors:** E. Riewe, E. Neubauer, A. C. Pfeifer, M. Schiltenwolf

**Affiliations:** 1 Academy of Specialized Health Professions (AGF), Neustadt a. d. W., Germany; 2 Center for Orthopedics, Trauma Surgery and Spinal Cord Injury, Heidelberg University Hospital, Heidelberg, Germany; The University of Tokyo Hospital, JAPAN

## Abstract

**Objective:**

10% of all individuals in Germany develop persistent symptoms due to nonspecific back pain (NSBP) causing up to 90% of direct and indirect expenses for health care systems. Evidence indicates a strong relationship between chronic nonspecific back pain and psychosocial risk factors. The Örebro Musculoskeletal Pain Screening Questionnaire (ÖMPSQ) and the German Heidelberger Kurzfragebogen Rückenschmerz (HKF-R 10) are deemed valid in prediction of persistent pain, functional loss or amount of sick leave. This study provides and discusses validity criteria for these questionnaires using ROC-curve analyses. Quality measurements included sensitivity and specificity, likelihood-ratio related test-efficiencies and clinical utility in regard to predictive values.

**Methods:**

265 patients recruited from primary and secondary care units completed both questionnaires during the same timeframe. From the total, 133 patients returned a 6-month follow-up questionnaire to assess the validity criteria for outcomes of pain, function and sick leave.

**Results:**

Based on heterogeneous cut-offs for the ÖMPSQ, sensitivity and specificity were moderate for outcome of pain (72%/75%). Very high sensitivity was observed for function (97%/57%) and high specificity for sick leave (63%/85%). The latter also applied to the HKF-R 10 (pain 50%/84%). Proportions between sensitivity and specificity were unbalanced except for the ÖMPSQ outcome of pain. Likelihood-ratios and positive predictive values ranged from low to moderate.

**Conclusion:**

Although the ÖMPSQ may be considered useful in identification of long-term functional loss or pain, over- and underestimation of patients at risk of chronic noncspecific back pain led to limited test-efficiencies and clinical utility for both questionnaires. Further studies are required to quantify the predictive validity of both questionnaires in Germany.

## Introduction

At present, chronic non-specific back pain (CNSBP) is considered a complex, multidimensional phenomenon, whereas origin and development is often traced back to psychosocial components. Conceptualised as “yellow flags” [1] these are deemed as moderate to strong predictors of chronicity due to acute back pain [2,3]. Prospective studies consistently illustrate the importance of yellow flags, in particular the transition from acute to chronic back pain [3–6].

About 10% to 15% of individuals with NSBP develop persistent problems that cause up to 90% of all direct and indirect expenses for health care systems in European countries [7,8,9]. Major costs result from sick leave and functional loss [10,11]. Even though, somatic interventions are common (surgery, medication, physiotherapy, occupational therapy), a combination with cognitive behavioural strategies has been proven more effective to treatment outcomes and lowering expenses. An Increasing number of publications support the superiority of these utilisations in a multimodal setting [12–15]. Numerous studies confirm the cost-effectiveness of multidisciplinary approaches with highlighting the importance of early identification of patients at risk of CNSBP [16,17,18].

The “Örebro Musculoskeletal Pain Screening Questionnaire” (ÖMPSQ) [19] and the German screening questionnaire “Heidelberger Kurzfragebogen Rückenschmerz” (HKF-R 10) [20] are designed to detect this patient group. Whereas, the HKF-R 10 predicts pain only and was not validated before, several single studies and reviews conceded at least moderate validity for the ÖMPSQ in prediction of sick listing, pain and function [21,22]. Commonly used by general practitioners, orthopaedic specialists/surgeons and physiotherapists in Germany, predictive validation for both questionnaires was missing during research and execution period of this study.

A recent publication tried to evaluate the predictive validity of these in a German sample [23]. It differs from the work of Linton and Boersma by its follow-up period (3 vs. 6 months) and usage of deviated items to assess deviated outcomes (e.g. long-term pain, functional loss and frequency of doctor’s advice). Furthermore, a direct comparison of both questionnaires for each of the outcomes was attempted. Results revealed high sensitivity and low specificity for the HKF-R 10 and inversed values for the ÖMPSQ. Concerning the discriminatory power, no recommendation for use was given due to over- and underestimation of patients at risk of CNSBP. Clinical utility has not been discussed.

We provided and discussed several validity criteria for the ÖMPSQ and the HKF-R 10 and aimed at a better understanding for the test properties of the ÖMPSQ. Quality Measurements included discriminatory power, test efficiencies in regards to likelihood-ratios and clinical utility for each of the questionnaires. No direct comparison was intended due to different items that assessed different outcomes (pain, function and sick leave for the ÖMPSQ and pain only for the HKF-R 10). To replicate foregoing results and to allow for the comparability to the work of Linton and Boersma, we employed similar methodological strategies. Because of Country specific differences according to the health care system, we slightly modified the eligibility criteria. For the HKF-R 10, specifications followed correspondence with the study authors.

## Methods

The ethical review committee of the University of Heidelberg approved this study in April 2012. Data were collected between May 2012 and February 2013 as well as between November 2012 and August 2013. Primary and secondary care units were asked to participate by written or oral enquiry. Orthopaedic specialists, rehabilitation facilities and private physiotherapy practices were selected to reflect upon the typical scenario for help-seeking patients. Data collection was not limited to a specific geographic region; moreover, units in several Federal States of Germany were chosen. After obtaining the units’ consent, sealed envelopes with questionnaires were sent out. Units were instructed to recruit patients who met the following eligibility criteria to participate: age > = 18 years; lower or upper back pain in first or second episode that requires further treatment; if in second episode, a required pain-free interval of 12 or more months between treatment of first and second episode; pain in acute or sub-acute stage (duration < three months); amount of sick leave < 6 month during the past year; adequate German language skills. The “required pain-free interval” criterion was used to preclude patients in a chronic/chronic recurrent state of NSBP [9].

Participating patients completed the questionnaire that combined all individual items of the HKF-R 10 and a German translation of the ÖMPSQ respectively. In line with specifications and approvement of the ethics review committee, this questionnaire contained two additional pages: a declaration of consent that must be signed by the participants and an information page to provide global information about this study. Futhermore, to prevent missing items an additional instructions page was also added to help patients in cases of uncertainty. Completed questionnaires were sent back to ER by the participating institutions. Following this, and after exclusion of questionnaires with non-signed declarations of consent random alphanumeric assignments were given to anonyms the individual data sets. The patients’ data and date of receipt were stored separately in electronic form. Six months’ later envelopes containing a follow-up questionnaire and a second envelope were sent to the participating patients directly. These did not contain any patient data except the alphanumeric assignment.

### Questionnaires

The ÖMPSQ is a widespread screening-questionnaire first developed in Sweden [24] to assess pain (5 items), functional ability (5 items), psychosocial (7 items) and work related variables (4 items) and is recognised in the German guideline for back pain [9]. The main intent was the early identification of psychosocial risk factors about sickness-related absenteeism. Its latest and modified version consists of 25 items [19,25]. Four items concerning sociodemographic information (age, gender, nationality, employment status) were excluded from scoring. Each item score ranged from 0 to 10 points except for three ranging from 1 to 10 points. For one of these 3 items with five categories (number of pain sites) the numbers were multiplied by two. To allow lower scores to indicate a lower risk, items 12, 16, 17, 21–25 have been inverted. Therefore, the linear total score ranged from 4 to 210 to identify individuals with low risk (< 90), medium risk (90–105) and high risk (> 105). Two work related items included an additional box „not working”(items 8, 17). If checked these were treated as a missing value [25]. In reference to Linton and Boersma in 2003 [19], the main outcome of sick leave was extended by adding functional ability and pain. A joining cut-off of 90 showed a sensitivity and specificity of 89%/65% for sick leave, 74%/79% for functional ability and 76%/70% for pain.

To evaluate the predictive validity for the ÖMPSQ we used a German translation developed by T. Kohlmann, C.O. Schmidt and M. Pfingsten in 2007. Development is based on international recommendations for self-reported measurements [26], whereas two independent translations and one re-translation were conducted [23]. It differs slightly from the original ÖMPSQ by including six categories for the number of pain sites and three work related items instead of two with the additional box „not working”(items 8, 16, 17). One sociodemographic item concerning nationality had been removed. The total score ranged from 4 to 212 respectively. Cut-offs regarding the original ÖMPSQ changed in consequence (< 91, 91–106, > 106).

The HKF-R 10 from 2006 [20] was intended for the early identification of psychosocial risk factors about persisting pain. This questionnaire has been also recognised by the German guideline for back pain [9]. It consists of 27 items assessing sociodemographic information (gender and graduation), pain intensity and duration (4 items), efficacy of massage (1 item), the Zung’s depression-scale (5 items) and the Kieler Schmerzinventar (KSI) consisting of catastrophising (5 items) and helpless-/hopelessness (9 items). Item 5 (average pain intensity during the last week) is excluded from scoring and should only be used as follow-up documentation. The non-linear total score was computed by summation of negative and positive sum products using an underlying multiple regression model. Individuals were divided into 5 categories: low risk (score < = -2.5), probably low risk (-2.5 < score < = 8), no prediction possible (8 < score < 28), probably high risk (28 < = score < 37), high risk (score > = 37). Sensitivity for outcome of pain was stated with 75% and specificity with 79%.

### Outcomes, Key-Variables and Follow-Up Questionnaire

About our study approach to replicate the ÖMPSQ in a German sample, we strictly followed the methodology of the original study in defining the key items and assessing the outcomes of pain, sick leave and function after six months. Regarding the HKF-R 10 (not validated during this study period), specifications set by EN were used to assess the outcome of pain.

The follow-up questionnaire was designed to assess the outcomes referenced above. It included all individual items of both questionnaires at first data collection to provide additional information if needed. Items to assess the outcomes read as follows.

Sick leave for the ÖMPSQ was assessed by adding self-reported days of sickness related absenteeism during the follow-up period (equivalent to item 6 but with a duration changed from 12 to 6 months). For function, the activity related items 21–25 were used.

Pain was assessed by pain intensity and pain frequency during the last 3 months (items 10+11).

For the HKF-R 10 item 5 considering the average pain intensity during the last week was used to assess outcome of pain. As mentioned previously, this item was excluded from scoring at baseline.

Outcomes for the ÖMPSQ were defined as follows.

Self-reported sick leave was divided into three groups: individuals with no sick leave (0 days), with short-term sick leave (1–30 days) and long-term sick leave (> 30 days). Defining sensitivity and specificity is prevented by using variables with non-dichotomous characteristic attributes. Therefore, ordinal or metric endpoints of outcome variables with more than two categories were artificially dichotomised to classify patients as “test-negative” (recovered) or “test-positive” (not recovered) only. Although loosing subgrouping information we dichotomised self-reported sick leave to divide patients with no sick leave (0 days) from those with short or long-term sick leave (> 0 days).

For function the scores of items 21–15 were summed (possible range 0–50) and a cut-off point at 45 was set to separate recovered from non-recovered patients (> = 45 recovered, < 45 not recovered). Pain for the ÖMPSQ was defined by multiplying items 10+11 (possible range 0–100). The cut-off was set at 17 to separate recovered from non-recovered patients (< 17 recovered, > = 17 not recovered). For the HKF-R 10 we used a cut-off set at 30 to separate recovered from non-recovered patients (< 30 recovered, > = 30 not recovered).

### Raw data handling and statistical analysis

For input and processing, such as computing of total scores and sub-scores MS Excel 2013 for Windows was used. Each data set contained items of both questionnaires and was divided into two partial data sets to handle raw data validation and further statistical analyses for each of the questionnaires. Missing values were not allowed for the HKF-R 10. For the ÖMPSQ we followed Linton’s approach to allow total score mean substitution [25]. Regarding the 3 checkboxes “not working” (treated as a missing value) we decided to allow a maximum of 3 substitutions, which is about 14% of 21 available items. In case of double entries on these items, an existing VAS-value overrides a checkbox value to avoid impending mean substitution. Raw data for each partial dataset was treated as valid if it had ended up in completeness (ÖMPSQ = 21 items, HKF-R 10 = 27 items). Invalid partial data sets were excluded.

IBM SPSS Statistics 20.0 and XLSTAT 2013 for Windows were used for calculations. The statistical significance level was set to 5% (α = 0.05) and the confidence interval (CI) to 95%.

Preliminary analyses of variance (ANOVA) were conducted to evaluate relationships between scoring levels at baseline and the characteristic attributes of the outcome variables. The level of mean differences between a low or a high total score at baseline (indicating a lower or a higher risk of CNSBP) and the outcome classification at follow-up (recovered/not recovered) indicates a missing or existing relationship.

To evaluate the discriminatory ability of both questionnaires, ROC curve analyses (Receiver Operation Characteristics) were conducted. The area under the curve (AUC) has been deemed as the most common measure of quality of information [27,28,29]. All additional information was provided by the corresponding tables of the ROC-curve coordinate points and its dependent values. Included measurements were cut-offs, sensitivity and specificity, prevalence’s, positive and negative likelihood-ratios/predictive values (PLR, NLR, LR, PPV, NPV, PV), accuracy of estimation (AAC) and sum of distributions. As recommended elsewhere, the Youden-Index (J = (Sensitivity+Specificity)-1) was used to determine the optimal (statistical) cut-off that proves the maximum potential effectiveness between sensitivity and specificity and affects the derived likelihood-ratios as well as the predictive values inside a sample [30,31]. The PPV and NPV (post-test probabilities) mainly depend on the prevalence of a disease (pre-test probabilities). In case of a sample’s prevalence that did not match the epidemiological one in the German population (10–15%), we followed statistical recommendations to re-calculate the PPV and NPV.

Classifications of test efficiencies concerning likelihood-ratios and its relationship to prevalence and predictive values are based on references that were found elsewhere [32,33,34]. Quality measurement of AUC’s and sum levels of sensitivity and specificity were classified for reference as follows: < 70% (sum < 140%) = low value; 70–80% (sum > = 140%, < 160%) = moderate value, 80–90% (sum 160%–180%) = good or high value, > 90% (sum > 180%) = very good or very high value (based on [32,34]). Tables 1 and 2 illustrate these classifications.

**Table 1:** Test efficiencies in regard to likelihood-ratios.

Positive Likelihood-Ratio	Negative Likelihood-Ratio	Test Efficiency
> 10	< 0,10	very high / very good
5–10	0.1–0.2	high /good
2–5	0.2–0.5	Moderate
1–2	0.5–1.0	Low

based on Mühlhauser and Höldke, 1999; Bender, 2001

**Table 2:** Dependencies between predictive values and prevalence.

Prevalence	Very high Test Efficiency		Moderate Test Efficiency	
	Sensitivity and Specificity = 0.95, PLR = 19		Sensitivity and Specificity = 0.7, PLR = 2.3	
	PPV	NPV	PPV	NPV
90%	99.4%	67.9%	95.5%	21%
50%	95.0%	95.0%	70%	70%
10%	67.9%	99.4%	21%	95%
1%	16.1%	> 99.9%	2.3%	99.6%
0.1%	1.9%	100%	0.2%	> 99.9%
0.01%	< 0.2%	100%	< 0.1%	100%

based on Mühlhauser and Höldke 1999; Bender, 2001; Grimes and Schulz, 2005

## Results

In total, 11 physiotherapy practices, 7 orthopaedic practitioners and 2 rehabilitation facilities agreed to participate. During the first data collection, 435 questionnaires were sent out of which 270 were completed. Unsigned declarations of consent (n = 4) and written withdrawal from study (n = 1) reduced the total number of questionnaires at baseline to n = 265. Raw data validation reduced the total amount of valid partial data sets at baseline to n = 241 for the ÖMPSQ and n = 242 for the HKF-R 10.

During follow-up, 133 questionnaires were returned. Missing mandatory case-boundaries of the total score at baseline and non-empty values of key variables at follow-up led to sub-samples with a reduced sample size. The sub-samples were as follows: HKF-R 10 pain (n = 128); ÖMPSQ pain and function (n = 122), sick leave (n = 108). Tables 3 and 4 depicts all sum distributions of data sets at baseline and at follow-up.

**Table 3:** Sum distributions of data sets in regard to outcome variables at baseline.

First data collection	ÖMPSQ	HKF-R 10
**N = 270**		
4 unsigned declarations of consent		
1 written withdrawal from study		
**N = 265**		
Raw data validation negative	24	23
**N**	**241**	**242**

**Table 4:** Sum distributions of data sets in regard to outcome variables at follow-up.

Follow-Up		ÖMPSQ		HKF-R 10
N = 133 of 265	Pain	Sick Leave	Fkt	Pain
Missing outcome variables (by list)	0	17	0	0
**N**	**133**	**116**	**133**	**133**
Valid data sets at baseline without bonds to outcome variables (by cases)	11	8	11	5
**N**	**122**	**108**	**122**	**128**

Total mean substitution was applied to 69 of 93 incomplete data sets for the ÖMPSQ. 46 substitutions were traced back to participants who indicated that they were currently “not working” by ticking the respective box for one or all of the three work related items. In 16 cases, neither the scales nor the checkboxes were ticked. It should be emphasised that this item group was often commented on by participants (n = 50 of 265).

### Baseline Characteristics

For the ÖMPSQ (n = 241) the mean age was 43 years, 65% of this distribution were female and 49% were full-time working. 63% of the participants reported multiple pain sites. Sickness-related absenteeism due to pain for more than a minimum of one day during the past year was reported by 37% of this sample. Pain duration of more than one week was reported by 94% of all participants and 15% reported pain durations of longer than 24 weeks. Mean pain intensity during the last week was 5.5 and 4.8 during the past 3 months. Mean pain frequency during the past three months was 6.

Distributions for the HKF-R 10 (n = 242) read as follow: 67% were female and 42% reported O-Level graduation. Pain duration of more than 8 days was indicated by 88% of the participants and multiple pain sites were stated by 59%. Pain relief after massage was reported by 52%. The mean pain intensity during the last week was 53.2 (range 0–100), and the best pain intensity was 27.9; mean accepted pain intensity after successful treatment was 11.6.

Tables A-E in S1 Data provide descriptive measures such as frequencies of completed questionnaires for each geographic region, total scores in outcome groups, tests on normal distribution and sociodemographic data with average scores of all individual items.

### Follow-Up—Means

Preliminary analyses of variance revealed a strong relationship between scores at baseline and the outcome attributes of pain, function and sick leave after six months (ÖMPSQ Pain, p < 0.001; HKF-R 10 Pain, p < 0.002; ÖMPSQ Function, p < 0.001; ÖMPSQ Sick leave, p < 0.001). Full ANOVA related calculations are outlined in Tables F-H in S1 Data.

### Follow-Up—Discriminative Abilities

As indicated in Table 5, ROC-curve AUC-levels for both questionnaires were statistically significant for each of the outcome groups. Results underscore the assumption that the discriminatory ability for each screening instrument is better than chance, with ÖMPSQ qualities ranging from moderate to good and a quality for the HKF-R 10 that was close to moderate. Generated ROC plots for each test variable can be found in Figures A-D in S2 Data.

**Table 5:** ROC-Analyses—Areas Under the Curve.

Test result Variables: Total Scores	TN	FP	TP	FN	Prevalence	Area	Std. Error^a^	Asymptotic Sig.^b^	Asymptotic 95% Confidence Interval	
									Lower Bound	Upper Bound
ÖMPSQ Pain	46	15	44	17	50%	0.785	0.041	0.000	0.706	0.865
ÖMPSQ Sick leave	58	10	25	15	37%	0.738	0.054	0.000	0.632	0.843
ÖMPSQ Functional Ability	33	25	62	2	52%	0.818	0.038	0.000	0.742	0.893
HKF-R10 Pain	32	6	45	45	70%	0.678	0.050	0.001	0.581	0.775

Each test result variable has at least one tie between the positive actual state group and the negative actual state group. Statistics may be biased.

Abbreviations: TN = True Negative; FP = False Positive; TP = True Positive; FN = False Negative

^a^. Under the nonparametric assumption

^b^. Null hypothesis: true area = 0.5

### Follow-Up—Predictive Validity Criteria

Table 6 shows all the predictive parameters for each outcome group. Underlying tables of ROC-curve coordinate points with extensive additional information are shown in Tables A-D in S2 Data.

**Table 6:** ROC-Analyses—Cut-off associated parameters of validity for each screening-tool taken from the ROC-curve coordinate point.

Instrument by Outcome	Cut-Off	Prevalence	Sensitivity	Specificity	PLR	NLR	PPV	NPV	ACC
	**84** ^a^	**50%** ^c^	**0.72**	**0.75**	**2.93**	**0.37**	**0.75**	**0.73**	**0.74**
ÖMPSQ		10% ^d^					0.25	0.96	
Pain	76 ^b^	50% ^c^	0.87	0.53	1.83	0.25	0.65	0.80	0.70
		10% ^d^					0.17	0.97	
	**92**	**37%** ^c^	**0.63**	**0.85**	**4.25**	**0.44**	**0.71**	**0.80**	**0.77**
		10% ^d^					0.32	0.95	
ÖMPSQ	84 ^a^	37% ^c^	0.70	0.71	2.38	0.43	0.58	0.80	0.70
Sick Leave		10% ^d^					0.21	0.96	
	76 ^b^	37% ^c^	0.80	0.44	1.43	0.45	0.46	0.79	0.57
		10% ^d^					0.14	0.95	
	**72**	**52%** ^c^	**0.97**	**0.57**	**2.25**	**0.06**	**0.71**	**0.94**	**0.78**
ÖMPSQ		10% ^d^					0.20	0.99	
Functional	84 ^a^	52% ^c^	0.70	0.76	2.91	0.39	0.76	0.70	0.73
Ability		10% ^d^					0.25	0.96	
	76 ^b^	50% ^c^	0.92	0.60	2.33	0.13	0.72	0.88	0.77
		10% ^d^					0.21	0.99	
	**46**	**70%** ^c^	**0.50**	**0.84**	**3.17**	**0.59**	**0.88**	**0.42**	**0.60**
		10% ^d^					0.26	0.94	
HKF-R 10	37 ^a^	70% ^c^	0.62	0.63	1.69	0.60	0.80	0.41	0.63
Pain		10% ^d^					0.16	0.94	
	20 ^b^	70% ^c^	0.86	0.29	1.20	0.50	0.74	0.46	0.69
		10% ^d^					0.12	0.95	

Values rounded; rows with optimal cut-offs in regard to sensitivity and specificity (calculated by the Youden-Index) are outlined bold

Abbreviations: PPV/NPV = positive/negative predictive value; PLR/NLR = positive/negative likelihoodratio; ACC = Accuracy

^a^. Cut-Off example with a balanced proportion between sensitivity and specificity

^b^. Cut-Off example with a sensitivity of 80% at minimum

^c^. prevalence within the sample

^d^. estimated prevalence in the German population

With a total score related cut-off of 84 in prediction of pain, moderate and balanced values were observed for the ÖMPSQ (sensitivity 72%, specificity 75%). Prediction of function showed very high sensitivity (97%) and low specificity (57%) with a total score cut-off of 71. In contrast and with inversed proportions, a total score cut-off of 92 predicted sick leave with low sensitivity (63%) and high specificity (85%). The same applied to prediction of pain for the HKF-R 10 (sensitivity 50%, specificity 84%) with a total score cut-off of 46. Predictive ACC levels for outcomes ranged between low for the HKF-R 10 in pain, moderate for the ÖMPSQ in pain and sick leave and close to high in function. As seen, our results revealed clearly lower cut-offs for the ÖMPSQ in outcomes of pain and function than by Linton and Boersma [19]. For the HKF-R 10 a cut-off clearly higher than the recommended one of 37 was observed [20].

As quality of LR’s rely on levels of sensitivity and specificity as well as on balanced proportions between both; the resulting test efficiencies could be classified as moderate at maximum for all outcomes of the questionnaires (see Tables 1 and 2). LR’s can be used to calculate the prevalence-dependent post-test probabilities of a disease (PPV and NPV). Post-test probabilities affect the clinical utility of a screening-tool by determining if further testing for a single patient due to test results is required or not. The samples’ prevalence appeared to be high and did not reflect the estimated prevalence rate of 10%–15% in the German population. Although PPV’s were all moderate for the ÖMPSQ (71–75%) and good for the HKF-R 10 (88%) within the sample, Table 6 highlights a subsequent fall-down below 33% if these were compared with a prevalence of 10% in the German population.

Table 6 also provides some total score cut-off examples (taken from tables of the ROC-curve coordinate points) to compare changes in quality measurements. If intention is to decrease false-negative results, a lowered cut-off may be chosen to increase sensitivity at cost of some specificity (rising amount of false-positive result). As seen, a cut-off of 20 for the HKF-R 10 and a joining one of 76 for all of the ÖMPSQ outcomes raised the sensitivity levels to at least 80%. Specificity became low in all these cases. Except for the ÖMPSQ’s prediction of function this was also true for the PLR’s and PPV’s. ACC levels were also lowered except for the HKF-R 10 with a value close to moderate. With an intention to find balanced values between sensitivity and specificity, a lowered cut-off of 37 for the HKF-R 10 and a joining one of 84 for the ÖMPSQ (based on prediction of pain) might be chosen. PLR’s, PPV’s and ACC levels changed in a similar way as in the previous cut-off example.

## Discussion

In reference to the work of Linton and Boersma and Neubauer et al. [19,20] we provided quality measurements of predictive validity for the ÖMPSQ and the HKF-R 10 in a German sample using ROC-Curve analyses. Preliminary ANOVA related results agree with findings of the referenced study [19] that prediction of pain, functional ability and amount of sickness related absenteeism after six months are influenced by psychosocial characteristics.

AUC-values of generated ROC-plots showed moderate and good discriminative levels for outcomes of the ÖMPSQ and a level close to moderate for outcome of the HKF-R 10. Findings for the ÖMPSQ agreed with several previous studies that also used ROC-Curve analyses to evaluate the predictive validity [21,22,35].

### Sensitivity and Specificity

As done by Linton and Boersma, we also provided cut-off examples to fit for high sensitivity or for balanced proportions between sensitivity and specificity. Additionally, and more important, optimised statistical cut-offs determined by the Youden-Index for each outcome group were provided.

Regarding these cut-offs and the intent of both questionnaires to reach high hit rates (sensitivity), agreement to Linton and Boersma exists in a moderate discriminative ability to identify patients at risk or no risk of persistent pain symptoms. Our results did not confirm their findings that tried to underline a high discriminatory power for sick listing.

Although sensitivity for outcome of function was very high, specificity was low and slightly above chance. This combination tends to overestimate patients at risk of CNSBP due to a rising number of false-positive results. An inversed proportion however, tends to underestimate the number of patients at risk by a rising amount of false-negatives. This is seen in prediction of sick leave for the ÖMPSQ and of pain for the HKF-R 10. In this regard, power of discrimination seems to be ambiguous and should be considered with care. To get around this, the provided cut-off example of 84 for all outcome groups should be favoured. This cut-off increases utilisation by unifying three different cut-offs, balances proportions and provides at least an overall moderate discriminative ability for the ÖMPSQ. Similarly, a cut-off of 37 for the HKF-R 10 that equals the original study should be recommended.

### Likelihood-Ratios, Test-Efficiencies and Predictive Values

Predictive validity of a screening-tool should include quality measurements of test efficiencies and clinical utility. Test-efficiencies regarding the intent of these questionnaires describe the power of LR’s to change the pre-test probability at risk of CNSBP to a sufficient PPV if the test result was positive. PPV’s will always be low if pre-test probabilities (such as the prevalence) are low—regardless of the power of LR’s. Compared with an established prevalence in the German population, inadequate representation of prevalence’s within our sample led to inadequate PPV’s. This is seen in our findings when trying to re-calculate the PV’s. In these cases, we encourage recommendations to use the prevalence independent LR’s instead. At least, these were moderate for all outcomes of both questionnaires.

### Weaknesses

Although strict eligibility criteria were chosen to minimise the amount of individuals’ that were in (recurrent) chronic state of NSBP at baseline, a potential selection bias could not be ruled out. This was indicated by reported pain duration for the ÖMPSQ of more than 24 weeks by 15% of all participants (43% in the original study). In addition, selection of participating patients by physicians and physiotherapists was maybe biased by their subjective view that patients were willing to cooperate and/or had an already existing higher risk of CNSBP. This may explain high prevalences within the sub-samples. Furthermore, by conducting ROC-Curve analyses that require dichotomised values, we lost subgrouping information that were originally available by graded classifications of both questionnaires.

Applying the methodological approach of Linton and Boersma in 2003, our study suffers from the same limitations. Some of the dependent variables have not been assessed by independent variables. This was true for the outcomes of function and pain. As already mentioned by Linton and Boersma, assessment by items that were part of the questionnaire itself (and were included in scoring) may inflate statistical results.

As seen, heterogeneous cut-offs within our sample for ÖMPSQ outcomes impede evaluation for examiners. These are traced back to different sub-sample sizes caused by negative raw data validation at baseline and/or missing replies to the key items at follow-up. The three work-related items were left empty several times. Uncertainties due to an inadequate reflection of the actual situation should be considered here. Furthermore, allowing for missing values (ticking checkboxes) led to an increase of potential dropouts [25]. The mean substitution with a quota of 14% in our population raises a statistical issue to mention. Aggravated by dependencies between the respective items, the advantage of easily applied mean substitution to complete the data sets and raising the sample size may have led to systematic statistical bias [36,37,38]. This is seen by ranges between 2 to almost 3 integers inside the bounds of several CI’s. However, and due to the design of the ÖMPSQ alternate modern statistical methods to handle missing values will be a difficult task in a triage setting. The HKF-R 10 was not affected by these kinds of bias due to prohibited substitution and usage of an outcome criterion that was excluded from scoring.

## Conclusions

Our results agreed with the referenced study in underlining a strong relationship between levels of pain, functional loss, sickness related absenteeism and existing yellow flags at earlier stage of non-specific back pain.

The ÖMPSQ showed moderate discriminative abilities with a joining cut-off of 84 and may be recognised for early identification of patients at risk of persistent pain and functional loss. The computed cut-off was lower than by Linton and Boersma in 2003. This might be explained by German specific differences in the health care system, ethical and socio-cultural backgrounds. Although underlined in the referenced study, high quality in prediction of sick leave could not be replicated. All determined cut-offs for the HKF-R 10 in prediction of pain revealed quality measurements that must be reviewed with care. Additionally, it seems to be questionable whether outcome of pain alone is a meaningful predictor of chronic non-specific back pain.

It should be stated that high sensitivity at cost of some specificity may be considered for both questionnaires in general. By adding progressive time pressure and need of saving expenses, costs for unnecessary and time-consuming examinations and treatments would be likely for individuals without risk. On the other hand, and in case of applied multimodal treatment models it could be argued that these costs after identification of individuals at risk of CNSBP (even on false-positive results) are in a much lower proportion compared to secondary costs arising from failed recognition of this group. A Swedish study demonstrated savings of about 90.000 Euro per head in a period of 7 years [16], whereas average daily costs for a three-week multimodal treatment in Germany are about 90–300 Euro [14,17]. Unfortunately, and as seen in our cut-off examples specificity results for all prediction outcomes fell near chance on high sensitivity if a lowered cut-off was chosen.

In the light of clinical utility, the use of predictive values should be avoided in our study population due to inadequate representations of positive post-test probabilities. Likelihood-ratios should be used instead, which were moderate for all outcomes of the questionnaires.

Furthermore, some scoring properties of the ÖMPSQ should be used with caution as they actually contain a potential risk of statistical bias while trying to evaluate this questionnaire. We recommend no admittance of missing values and double entries. That means control of the filled out questionnaire and explanation for some items in addition. This however, will become a difficult task for examiners in regard of existing time pressure.

In summary and in inclusion of all mentioned aspects of the predictive validity criteria, recommendation for use of these questionnaires is limited. Low to moderate discriminative power and moderate clinical utility will require additional tests in a triage setting to rule-in chronicity due to long-term pain, functional loss and the amount of sick leave.

Concerning the use of total score dependent cut-offs for both screening-tools, some authors critically reviewed and discussed this approach for the ÖMPSQ. Instead, calculation of correlations and weights between single items or item groups and an external outcome criterion were recommended. In line with usage of prediction rules, a more specific risk profile was suggested that could also be employed to determine the treatment strategies [39]. However, we sought for validation of the ÖMPSQ in reference to the work of Linton and Boersma in 2003. Although we second the recommendations above, scoring properties as well as defining the outcome parameters should also be reconsidered prior to avoid uncertainty and statistical bias in general.

## Supporting Information

S1 Data (DOCX)

S2 Data (DOCX)
