# Supplementary material for: Predicting Persistent Back Symptoms by Psychosocial Risk Factors: Validity Criteria for the ÖMPSQ and the HKF-R 10 in Germany
Source: PLoS One. 2016 Jul 21;11(7):e0158850. doi: 10.1371/journal.pone.0158850 (PMC4956238; doi:10.1371/journal.pone.0158850)
Supplement: S1 Data — (DOCX) [file pone.0158850.s001.docx]

**S1 Table A. regional distributions of received questionnaires at baseline (n=265).**

| Postal code (prefix) | Districts | Federal State | Sum | Percentage^a^ |
| --- | --- | --- | --- | --- |
| 7 | Siegen-Wittgenstein | NRW | 209 | 78.9% |
| 35 | Lahn-Dill Kreis | Hessen | 30 | 11.3% |
| 68 | Mannheim | BW | 7 | 2.6% |
| 74 | Heilbronn | BW | 5 | 1.9% |
| 22 | Hamburg | Hamburg | 3 | 1.1% |
| 56 | Westerwaldkreis | RP | 3 | 1.1% |
| 77 | Ortenaukreis | BW | 3 | 1.1% |
| 51 | Oberbergischer Kreis | NRW | 2 | 0.8% |
| 53 | Bonn. Stadt | NRW | 1 | 0.4% |
| 59 | Hochsauerlandkreis | NRW | 1 | 0.4% |
| - | none - eMail only | - | 1 | 0.4% |
|  |  | Sum: | 265 | 100.0% |

Abbreviations: NRW=Nordrhein-Westfalen; BW=Baden-Württemberg; RP= Rheinland-Pfalz

^a^ rounded

**S1 Table B. Total scores in outcome groups at baseline – descriptive statistics.**

| Scores at baseline | N | Range | Minimum | Maximum | Mean | | Std. Deviation |
| --- | --- | --- | --- | --- | --- | --- | --- |
|  | Statistic | Statistic | Statistic | Statistic | Statistic | Std. Error | Statistic |
| ÖMPSQ Pain | 122 | 124 | 36 | 160 | 85.07 | 2.345 | 25.902 |
| ÖMPSQ Sick Leave | 108 | 124 | 36 | 160 | 82.41 | 2.363 | 24.561 |
| ÖMPSQ Functional Ability | 122 | 124 | 36 | 160 | 85.07 | 2.345 | 25.902 |
| HKFR10 Pain | 128 | 167.2 | -36.6 | 130.6 | 41.62 | 2.575 | 29.135 |

**S1 Table C. Total scores in outcome groups at baseline - tests of normal distribution.**

| Scores at baseline | N | Kolmogorov-Smirnov | | Shapiro-Wilk | | Anderson-Darling | |
| --- | --- | --- | --- | --- | --- | --- | --- |
|  |  | Statistic | Significance | Statistic | Significance | Statistic | Significance |
| ÖMPSQ Pain | 122 | 0.076 | 0.500 | 0.983 | 0.116 | 0.496 | 0.210 |
| ÖMPSQ Sick Leave | 108 | 0.069 | 0.681 | 0.982 | 0.149 | 0.445 | 0.284 |
| ÖMPSQ Functional Ability | 122 | 0.075 | 0.500 | 0.983 | 0.116 | 0.499 | 0.211 |
| HKFR10 Pain | 128 | 0.057 | 0.796 | 0.991 | 0.551 | 0.512 | 0.195 |

**S1 Table D. HKF-R 10 scores, means and standard deviations at baseline.**

|  |  | Baseline | |
| --- | --- | --- | --- |
|  |  | (n=242 of 265) | |
| Item | Scale/Format | Mean/% | SD/Range |
| 1 | Gender (male/female) |  |  |
|  | Female | 66.90% |  |
| 2 | School -leaving qualification |  |  |
|  | No qualification/ Secondary school | 20.20% |  |
|  | O-Level | 42.10% |  |
|  | Technical college certificate | 12% |  |
|  | A-Level/Technical College | 16.90% |  |
|  | University degree/Postgraduate (Ph.D.) | 8.70% |  |
| 3 | Duration of pain longer than 8 days (Yes/No) |  |  |
|  | Yes | 88.40% |  |
| 4 | Other pain sites than low back pain (Yes/No) |  |  |
|  | Yes | 59.10% |  |
| 5 | Pain intensity during the past week (0-100) | 53.14 | 22.129 |
| 6 | Pain intensity during the past week in best stage (0-100) | 27.85 | 21.138 |
| 7 | Expected pain intensity after successful treatment (0-100) | 11.57 | 13.965 |
| 8 | Pain relief after massage (Yes/No) |  |  |
|  | Yes | 51.70% |  |
| ***9*** | ***If you were conscious of pain during the last 14 days,***  ***How often did you have the following thoughts and feelings?*** |  |  |
| 9a | What’s at the bottom of it? | 3.483 | 1.753 |
| 9b | Why do I have to stand this burden? | 1.723 | 1.729 |
| 9c | I almost thing they won’t go away. | 2.785 | 1.861 |
| 9d | This bad pain spoils everything! | 2.293 | 1.849 |
| 9e | What does it means? | 2.124 | 1.834 |
| 9f | I hopefully do not have cancer? | 0.979 | 1.638 |
| 9g | I do not stand it any longer! | 1.661 | 1.645 |
| 9h | Whether I suffer from the same sickness than… | 0.851 | 1.506 |
| 9j | It doesn’t get better. | 2.314 | 1.736 |
| 9k | Ohhhh, the whole day is spoiled again! | 1.707 | 1.797 |
| 9l | Life with this pain is almost not worth-living! | 1.033 | 1.564 |
| 9m | What can I do if it gets worse again? | 2.103 | 1.702 |
| 9n | For how long I have to bear this pain? | 2.384 | 1.789 |
| 9o | It’s not a bad sickness behind this pain? | 1.360 | 1.738 |
|  |  |  |  |
| ***10*** | ***How was your condition within the last 14 days?*** |  |  |
| 10a | I feel down or depressed. | 0.769 | 0.770 |
| 10b | I cry a lot or I feel like crying. | 0.409 | 0.724 |
| 10c | It takes time to fall asleep. | 1.099 | 0.959 |
| 10d | I’m restless. | 0.917 | 0.921 |
| 10e | I still enjoy doing things I liked before. | 1.893 | 0.967 |

**S1 Table E. ÖMPSQ scores, means and standard deviations at baseline.**

|  | | | Baseline | |
| --- | --- | --- | --- | --- |
|  | | | (n=241 of 265) | |
| Item ^a^ | | Scale/Format | Mean/% | SD/Range |
| 1 | Birth (blank) | | 1969.9 | 20-81 |
| 2 | Gender (male/female) | |  |  |
|  | Female | | 65.10% |  |
| 3 | Current employment status | |  |  |
|  | employed – full-time | | 49% |  |
|  | employed – more than half-time | | 14.90% |  |
|  | employed – less than half-time | | 14.10% |  |
|  | Not employed – housewife/househusband | | 10.40% |  |
|  | Not employed – out-of-school education/re-education | | 3.30% |  |
|  | Not employed – unemployed | | 0.80% |  |
|  | Not employed – disability pension | | 0.40% |  |
|  | Not employed – retirement annuity | | 2.90% |  |
|  | Not employed – others | | 4.10% |  |
| 4 | Where do you have pain (6 possible numbers of pain sites) | |  |  |
|  | > 1 | | 62.65% |  |
| 5 | Sick leave due to pain (past 12 months) | |  |  |
|  | 0 days | | 63.07% |  |
|  | 1-30 days | | 28.63% |  |
|  | > 30 days | | 8.9% |  |
| 6 | Duration suffering from pain (weeks) | | 5.43 | 2.411 |
|  | >1 week | | 93.77% |  |
|  | >24 weeks | | 14.93% |  |
| 7 | Heavy or monotonous work | | 4.834 | 2.466 |
| 8 | Pain intensity during the past week | | 5.494 | 2.070 |
| 9 | Average pain intensity during the past 3 months | | 4.805 | 2.004 |
| 10 | Average pain frequency during past 3 months | | 5.967 | 2.628 |
| 11 | Ability to decrease pain on an average day | | 4.556 | 2.375 |
| 12 | Anxiety and tenseness during past week | | 5.851 | 2.45 |
| 13 | Intensity of feeling depressed during past week | | 4.037 | 2.866 |
| 14 | Subjective view that pain becomes persistent | | 5.473 | 2.717 |
| 15 | Subjective view of being at work again after six months | | 8.041 | 2.435 |
| 16 | Subjective job satisfaction | | 6.556 | 2.302 |
| 17 | Belief that physical activity makes pain worse | | 5.261 | 3.243 |
| 18 | Belief that increasing pain indicates to stop current activity | | 6.124 | 3.100 |
| 19 | Belief that normal work should not be done with present pain | | 3.311 | 3.137 |
| 20 | Ability to do light work for an hour | | 8.282 | 2.383 |
| 21 | Ability to walk for an hour | | 8.071 | 2.719 |
| 22 | Ability to do ordinary houshold chores | | 7.689 | 2.304 |
| 23 | Ability to do shopping | | 7.917 | 2.429 |
| 24 | Ability to sleep at night | | 6.432 | 2.729 |

^a^ Item count of the German translation (shifts one step down compared to the original ÖMPSQ)

**S1 Table F. ONEWAY ANOVA: Test of Homogeneity of Variances.**

| Total scores at baseline  grouped by outcome variables at follow-up | Levenne-Statistic | df1 | df2 | Significance |
| --- | --- | --- | --- | --- |
| ÖMPSQ Pain | 0.751 | 1 | 120 | 0.388 |
| ÖMPSQ Sick Leave (dichotomised) | 3.521 | 1 | 106 | 0.063 |
| ÖMPSQ Sick Leave | 1.809 | 2 | 105 | 0.169 |
| ÖMPSQ Functional Ability | 0.007 | 1 | 120 | 0.933 |
| HKFR10 Pain | 2.198 | 1 | 126 | 0.141 |

Abbreviations: df1, df2 = degrees of freedom

**S1 Table G. ONEWAY ANOVA.**

| Total scores at baseline  grouped by outcome variables at follow-up | | Sum of Squares | Df | Mean Square | F | Significance |
| --- | --- | --- | --- | --- | --- | --- |
| ÖMPSQ Pain | Between Groups | 19616.467 | 1 | 19616.467 | 38.236 | 0.000 |
|  | Within Groups | 61563.869 | 120 | 513.032 |  |  |
|  | Total | 81180.336 | 121 |  |  |  |
| ÖMPSQ Sick Leave | Between Groups | 10234.709 | 1 | 10234.709 | 19.974 | 0.000 |
| (dichotomised) | Within Groups | 54313.365 | 106 | 512.390 |  |  |
|  | Total | 64548.074 | 107 |  |  |  |
| ÖMPSQ Sick Leave | Between Groups | 15315.868 | 2 | 7657.934 | 16.332 | 0.000 |
|  | Within Groups | 49232.206 | 105 | 468.878 |  |  |
|  | Total | 64548.074 | 107 |  |  |  |
| ÖMPSQ Functional Ability | Between Groups | 24436.959 | 1 | 24436.959 | 51.679 | 0.000 |
|  | Within Groups | 56743.377 | 120 | 472.861 |  |  |
|  | Total | 81180.336 | 121 |  |  |  |
| HKFR10 Pain | Between Groups | 7718.372 | 1 | 7718.372 | 9.717 | 0.002 |
|  | Within Groups | 100086.423 | 126 | 794.337 |  |  |
|  | Total | 107804.795 | 127 |  |  |  |

Abbreviations: Df = Degrees of freedom

**S1 Table H. ONEWAY ANOVA: Descriptive Statistics**

| Total scores at baseline  grouped by attributes of outcome variables at follow-up | | | N | Mean | Percent | Std. Deviation | Std. Error | 95%-Confidence Interval for Mean | | Minimum | Maximum | Between-Component Variance |
| --- | --- | --- | --- | --- | --- | --- | --- | --- | --- | --- | --- | --- |
|  |  |  |  |  |  |  |  | Lower Bound | Upper Bound |  |  |  |
| ÖMPSQ Pain | 1_Recovered | | 61 | 72.39 | 50.0 | 21.229 | 2.718 | 66.96 | 77.83 | 36 | 127 |  |
|  | 2_Not recovered | | 61 | 97.75 | 50.0 | 23.987 | 3.071 | 91.61 | 103.90 | 50 | 160 |  |
|  | Total | | 122 | 85.07 | 100 | 25.902 | 2.345 | 80.43 | 89.72 | 36 | 160 |  |
|  | Model | Fixed Effects |  |  |  | 22.650 | 2.051 | 81.01 | 89.13 |  |  |  |
|  |  | Random Effects |  |  |  |  | 12.680 | -76.05 | 246.19 |  |  | 313.171 |
| ÖMPSQ Sick Leave  (dichotomised) | 1_No sick leave | | 68 | 74.94 | 63.0 | 19.779 | 2.399 | 70.15 | 79.73 | 39 | 138 |  |
|  | 2_Sick leave | | 40 | 95.10 | 37.0 | 26.843 | 4.244 | 86.52 | 103.68 | 36 | 160 |  |
|  | Total | | 108 | 82.41 | 100 | 24.561 | 2.363 | 77.72 | 87.09 | 36 | 160 |  |
|  | Model | Fixed Effects |  | 22.636 |  | 2.178 | 78.09 | 86.73 |  |  |  |  |
|  |  | Random Effects |  |  |  | 10.380 | -49.48 | 214.29 |  |  |  | 193.017 |
| ÖMPSQ Sick Leave | 1_No sick leave | | 68 | 74.94 | 63.0 | 19.779 | 2.399 | 70.15 | 79.73 | 39 | 138 |  |
|  | 2_1-30 days | | 33 | 89.91 | 30.6 | 26.149 | 4.552 | 80.64 | 99.18 | 36 | 160 |  |
|  | 3_More than 30 days | | 7 | 119.57 | 6.5 | 13.782 | 5.209 | 106.82 | 132.32 | 101 | 136 |  |
|  | Total | | 108 | 82.41 | 100 | 24.561 | 2.363 | 77.72 | 87.09 | 36 | 160 |  |
|  | Model | Fixed Effects |  | 21.654 |  | 2.084 | 78.28 | 86.54 |  |  |  |  |
|  |  | Random Effects |  |  |  | 11.589 | 32.54 | 132.27 |  |  |  | 263.103 |
| ÖMPSQ Functional Ability | 1_Recovered | | 58 | 70.21 | 47.5 | 21.552 | 2.830 | 64.54 | 75.87 | 36 | 114 |  |
|  | 2_Not recovered | | 64 | 98.55 | 52.5 | 21.919 | 2.740 | 93.07 | 104.02 | 64 | 160 |  |
|  | Total | | 122 | 85.07 | 100 | 25.902 | 2.345 | 80.43 | 89.72 | 36 | 160 |  |
|  | Model | Fixed Effects |  | 21.745 |  | 1.969 | 81.18 | 88.97 |  |  |  |  |
|  |  | Random Effects |  |  |  | 14.186 | -95.18 | 265.33 |  |  |  | 393.807 |
| HKFR10 Pain | 1_Recovered | | 38 | 29.674 | 29.7 | 24.4754 | 3.9704 | 21.629 | 37.719 | -22.5 | 93.9 |  |
|  | 2_Not recovered | | 90 | 46.670 | 70.3 | 29.5893 | 3.1190 | 40.473 | 52.867 | -36.6 | 130.6 |  |
|  | Total | | 128 | 41.624 | 100 | 29.1351 | 2.5752 | 36.528 | 46.720 | -36.6 | 130.6 |  |
|  | Model | Fixed Effects |  | 28.1840 |  | 2.4911 | 36.694 | 46.554 |  |  |  |  |
|  |  | Random Effects |  |  |  | 9.0379 | -73.214 | 156.462 |  |  |  | 129.5726 |
