# Supplementary material for: Predicting Persistent Back Symptoms by Psychosocial Risk Factors: Validity Criteria for the ÖMPSQ and the HKF-R 10 in Germany
Source: PLoS One. 2016 Jul 21;11(7):e0158850. doi: 10.1371/journal.pone.0158850 (PMC4956238; doi:10.1371/journal.pone.0158850)
Supplement: S2 Data — (DOCX) [file pone.0158850.s002.docx]

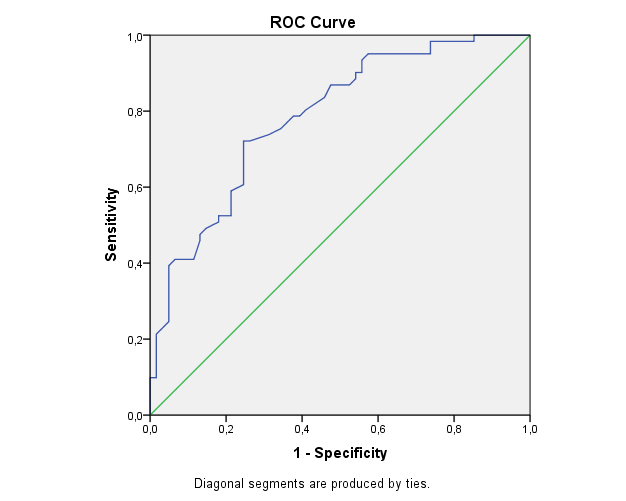

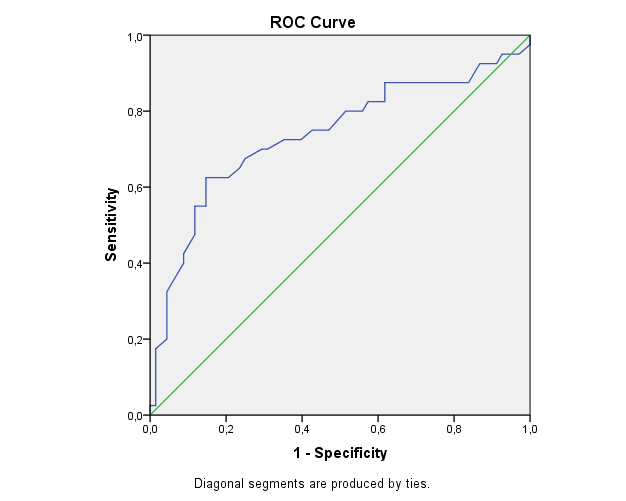


S2 Fig B. ROC-Plot for the ÖMPSQ’s total score ability to predict sick leave at 6 months’ follow-up.

S2 Fig A. ROC-Plot for the ÖMPSQ´s total score ability to predict Pain at 6 months´follow-up.


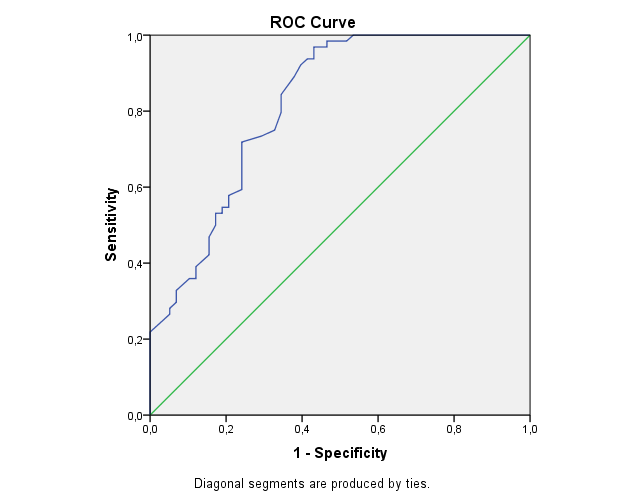

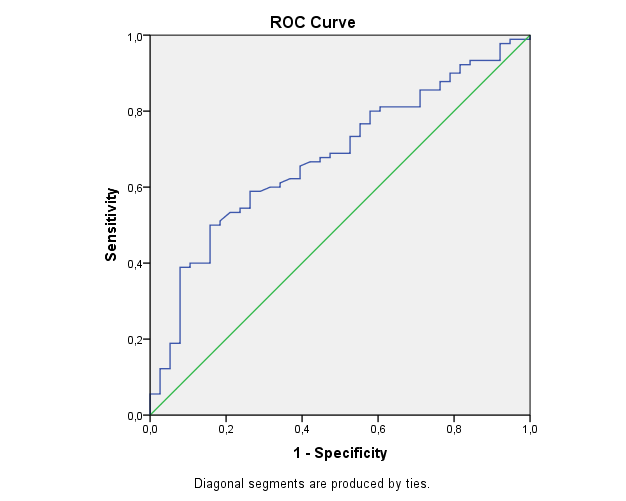


S2 Fig C. ROC-Plot for the ÖMPSQ’s total score ability to predict functional ability at 6 months´ follow-up.

S2 Fig D. ROC-Plot for the HKF-R 10’s total score ability to predict pain at 6 months´ follow-up.

**S2 Table A. Excerpts of the ROC-curve coordinates: ÖMPSQ outcome of pain.**

| ÖMPSQ Score Cut-Offs  (n=122) | Sensitivity | LB (95%) | UB (95%) | Specificity | LB (95%) | UB (95%) | PPV | NPV | PLR | NLR | TP | TN | FP | FN | J | ACC |
| --- | --- | --- | --- | --- | --- | --- | --- | --- | --- | --- | --- | --- | --- | --- | --- | --- |
| 68,000 | 0,918 | 0,817 | 0,968 | 0,443 | 0,325 | 0,567 | 0,622 | 0,844 | 1,647 | 0,185 | 56 | 27 | 34 | 5 | 0,680 | 1,361 |
| 69,000 | 0,902 | 0,797 | 0,957 | 0,443 | 0,325 | 0,567 | 0,618 | 0,818 | 1,618 | 0,222 | 55 | 27 | 34 | 6 | 0,672 | 1,344 |
| 70,000 | 0,902 | 0,797 | 0,957 | 0,459 | 0,340 | 0,583 | 0,625 | 0,824 | 1,667 | 0,214 | 55 | 28 | 33 | 6 | 0,680 | 1,361 |
| 72,000 | 0,885 | 0,778 | 0,946 | 0,459 | 0,340 | 0,583 | 0,621 | 0,800 | 1,636 | 0,250 | 54 | 28 | 33 | 7 | 0,672 | 1,344 |
| 74,000 | 0,869 | 0,758 | 0,934 | 0,475 | 0,356 | 0,598 | 0,624 | 0,784 | 1,656 | 0,276 | 53 | 29 | 32 | 8 | 0,672 | 1,344 |
| 75,000 | 0,869 | 0,758 | 0,934 | 0,492 | 0,371 | 0,614 | 0,631 | 0,789 | 1,710 | 0,267 | 53 | 30 | 31 | 8 | 0,361 | 0,680 |
| 76,000 | 0,869 | 0,758 | 0,934 | 0,525 | 0,402 | 0,644 | 0,646 | 0,800 | 1,828 | 0,250 | 53 | 32 | 29 | 8 | 0,393 | 0,697 |
| 77,000 | 0,836 | 0,721 | 0,910 | 0,541 | 0,417 | 0,660 | 0,646 | 0,767 | 1,821 | 0,303 | 51 | 33 | 28 | 10 | 0,377 | 0,689 |
| 78,000 | 0,803 | 0,685 | 0,885 | 0,590 | 0,465 | 0,704 | 0,662 | 0,750 | 1,960 | 0,333 | 49 | 36 | 25 | 12 | 0,393 | 0,697 |
| 79,000 | 0,787 | 0,667 | 0,872 | 0,607 | 0,481 | 0,719 | 0,667 | 0,740 | 2,000 | 0,351 | 48 | 37 | 24 | 13 | 0,393 | 0,697 |
| 80,000 | 0,787 | 0,667 | 0,872 | 0,623 | 0,497 | 0,734 | 0,676 | 0,745 | 2,087 | 0,342 | 48 | 38 | 23 | 13 | 0,410 | 0,705 |
| 81,000 | 0,754 | 0,632 | 0,845 | 0,656 | 0,530 | 0,762 | 0,687 | 0,727 | 2,190 | 0,375 | 46 | 40 | 21 | 15 | 0,410 | 0,705 |
| 82,000 | 0,738 | 0,614 | 0,832 | 0,689 | 0,563 | 0,791 | 0,703 | 0,724 | 2,368 | 0,381 | 45 | 42 | 19 | 16 | 0,426 | 0,713 |
| 83,000 | 0,721 | 0,597 | 0,818 | 0,738 | 0,614 | 0,832 | 0,733 | 0,726 | 2,750 | 0,378 | 44 | 45 | 16 | 17 | 0,459 | 0,730 |
| **84,000** | **0,721** | **0,597** | **0,818** | **0,754** | **0,632** | **0,845** | **0,746** | **0,730** | **2,933** | **0,370** | **44** | **46** | **15** | **17** | **0,475** | **0,738** |
| 85,000 | 0,639 | 0,513 | 0,748 | 0,754 | 0,632 | 0,845 | 0,722 | 0,676 | 2,600 | 0,478 | 39 | 46 | 15 | 22 | 0,393 | 0,697 |
| 88,000 | 0,607 | 0,481 | 0,719 | 0,754 | 0,632 | 0,845 | 0,712 | 0,657 | 2,467 | 0,522 | 37 | 46 | 15 | 24 | 0,361 | 0,680 |
| 89,000 | 0,590 | 0,465 | 0,704 | 0,787 | 0,667 | 0,872 | 0,735 | 0,658 | 2,769 | 0,521 | 36 | 48 | 13 | 25 | 0,377 | 0,689 |
| 90,000 | 0,557 | 0,433 | 0,675 | 0,787 | 0,667 | 0,872 | 0,723 | 0,640 | 2,615 | 0,563 | 34 | 48 | 13 | 27 | 0,344 | 0,672 |
| 91,000 | 0,541 | 0,417 | 0,660 | 0,787 | 0,667 | 0,872 | 0,717 | 0,632 | 2,538 | 0,583 | 33 | 48 | 13 | 28 | 0,328 | 0,664 |
| 92,000 | 0,525 | 0,402 | 0,644 | 0,787 | 0,667 | 0,872 | 0,711 | 0,623 | 2,462 | 0,604 | 32 | 48 | 13 | 29 | 0,311 | 0,656 |
| 93,000 | 0,525 | 0,402 | 0,644 | 0,803 | 0,685 | 0,885 | 0,727 | 0,628 | 2,667 | 0,592 | 32 | 49 | 12 | 29 | 0,328 | 0,664 |
| 94,000 | 0,525 | 0,402 | 0,644 | 0,820 | 0,703 | 0,897 | 0,744 | 0,633 | 2,909 | 0,580 | 32 | 50 | 11 | 29 | 0,344 | 0,672 |
| 95,000 | 0,508 | 0,386 | 0,629 | 0,820 | 0,703 | 0,897 | 0,738 | 0,625 | 2,818 | 0,600 | 31 | 50 | 11 | 30 | 0,328 | 0,664 |
| 96,000 | 0,492 | 0,371 | 0,614 | 0,852 | 0,740 | 0,922 | 0,769 | 0,627 | 3,333 | 0,596 | 30 | 52 | 9 | 31 | 0,344 | 0,672 |
| 100,000 | 0,475 | 0,356 | 0,598 | 0,869 | 0,758 | 0,934 | 0,784 | 0,624 | 3,625 | 0,604 | 29 | 53 | 8 | 32 | 0,344 | 0,672 |
| 101,000 | 0,459 | 0,340 | 0,583 | 0,869 | 0,758 | 0,934 | 0,778 | 0,616 | 3,500 | 0,623 | 28 | 53 | 8 | 33 | 0,328 | 0,664 |
| 102,000 | 0,410 | 0,296 | 0,535 | 0,885 | 0,778 | 0,946 | 0,781 | 0,600 | 3,571 | 0,667 | 25 | 54 | 7 | 36 | 0,295 | 0,648 |
| 103,000 | 0,410 | 0,296 | 0,535 | 0,918 | 0,817 | 0,968 | 0,833 | 0,609 | 5,000 | 0,643 | 25 | 56 | 5 | 36 | 0,328 | 0,664 |
| 104,000 | 0,410 | 0,296 | 0,535 | 0,934 | 0,837 | 0,978 | 0,862 | 0,613 | 6,250 | 0,632 | 25 | 57 | 4 | 36 | 0,344 | 0,672 |
| 106,000 | 0,393 | 0,281 | 0,519 | 0,951 | 0,858 | 0,988 | 0,889 | 0,611 | 8,000 | 0,638 | 24 | 58 | 3 | 37 | 0,344 | 0,672 |
| 108,000 | 0,361 | 0,252 | 0,487 | 0,951 | 0,858 | 0,988 | 0,880 | 0,598 | 7,333 | 0,672 | 22 | 58 | 3 | 39 | 0,656 | 1,311 |

Abbreviations: LB=Lower Bound; UB=Upper Bound; TP=True Positive; TN=True Negative; FP=False Positive; FN=False Negative; J=Youden-Index; ACC=Accuracy; row with optimal statistical cut-off by Youden-Index is outlined bold

**S2 Table B. Excerpts of the ROC-curve coordinates: ÖMPSQ outcome of sick leave.**

| ÖMPSQ Score Cut-Offs  (n=108) | Sensitivity | LB (95%) | UB (95%) | Specificity | LB (95%) | UB (95%) | PPV | NPV | PLR | NLR | TP | TN | FP | FN | J | ACC |
| --- | --- | --- | --- | --- | --- | --- | --- | --- | --- | --- | --- | --- | --- | --- | --- | --- |
| 65,000 | 0,875 | 0,733 | 0,949 | 0,338 | 0,237 | 0,457 | 0,438 | 0,821 | 1,322 | 0,370 | 35 | 23 | 45 | 5 | 0,213 | 0,537 |
| 67,000 | 0,875 | 0,733 | 0,949 | 0,368 | 0,263 | 0,487 | 0,449 | 0,833 | 1,384 | 0,340 | 35 | 25 | 43 | 5 | 0,243 | 0,556 |
| 68,000 | 0,875 | 0,733 | 0,949 | 0,382 | 0,276 | 0,501 | 0,455 | 0,839 | 1,417 | 0,327 | 35 | 26 | 42 | 5 | 0,257 | 0,565 |
| 70,000 | 0,850 | 0,704 | 0,932 | 0,382 | 0,276 | 0,501 | 0,447 | 0,813 | 1,376 | 0,392 | 34 | 26 | 42 | 6 | 0,232 | 0,556 |
| 72,000 | 0,825 | 0,676 | 0,915 | 0,382 | 0,276 | 0,501 | 0,440 | 0,788 | 1,336 | 0,458 | 33 | 26 | 42 | 7 | 0,207 | 0,546 |
| 74,000 | 0,825 | 0,676 | 0,915 | 0,412 | 0,303 | 0,531 | 0,452 | 0,800 | 1,403 | 0,425 | 33 | 28 | 40 | 7 | 0,237 | 0,565 |
| 75,000 | 0,825 | 0,676 | 0,915 | 0,426 | 0,316 | 0,545 | 0,458 | 0,806 | 1,438 | 0,410 | 33 | 29 | 39 | 7 | 0,251 | 0,574 |
| 76,000 | 0,800 | 0,649 | 0,897 | 0,441 | 0,330 | 0,559 | 0,457 | 0,789 | 1,432 | 0,453 | 32 | 30 | 38 | 8 | 0,241 | 0,574 |
| 77,000 | 0,800 | 0,649 | 0,897 | 0,485 | 0,371 | 0,602 | 0,478 | 0,805 | 1,554 | 0,412 | 32 | 33 | 35 | 8 | 0,285 | 0,602 |
| 78,000 | 0,750 | 0,596 | 0,859 | 0,529 | 0,412 | 0,643 | 0,484 | 0,783 | 1,594 | 0,472 | 30 | 36 | 32 | 10 | 0,279 | 0,611 |
| 79,000 | 0,750 | 0,596 | 0,859 | 0,559 | 0,441 | 0,670 | 0,500 | 0,792 | 1,700 | 0,447 | 30 | 38 | 30 | 10 | 0,309 | 0,630 |
| 80,000 | 0,750 | 0,596 | 0,859 | 0,574 | 0,455 | 0,684 | 0,508 | 0,796 | 1,759 | 0,436 | 30 | 39 | 29 | 10 | 0,324 | 0,639 |
| 81,000 | 0,725 | 0,570 | 0,839 | 0,603 | 0,484 | 0,711 | 0,518 | 0,788 | 1,826 | 0,456 | 29 | 41 | 27 | 11 | 0,328 | 0,648 |
| 82,000 | 0,725 | 0,570 | 0,839 | 0,647 | 0,528 | 0,750 | 0,547 | 0,800 | 2,054 | 0,425 | 29 | 44 | 24 | 11 | 0,372 | 0,676 |
| 83,000 | 0,700 | 0,544 | 0,819 | 0,691 | 0,573 | 0,788 | 0,571 | 0,797 | 2,267 | 0,434 | 28 | 47 | 21 | 12 | 0,391 | 0,694 |
| 84,000 | 0,700 | 0,544 | 0,819 | 0,706 | 0,588 | 0,801 | 0,583 | 0,800 | 2,380 | 0,425 | 28 | 48 | 20 | 12 | 0,406 | 0,704 |
| 85,000 | 0,675 | 0,519 | 0,799 | 0,750 | 0,634 | 0,838 | 0,614 | 0,797 | 2,700 | 0,433 | 27 | 51 | 17 | 13 | 0,425 | 0,722 |
| 88,000 | 0,650 | 0,494 | 0,779 | 0,765 | 0,650 | 0,850 | 0,619 | 0,788 | 2,763 | 0,458 | 26 | 52 | 16 | 14 | 0,415 | 0,722 |
| 89,000 | 0,625 | 0,470 | 0,758 | 0,794 | 0,682 | 0,874 | 0,641 | 0,783 | 3,036 | 0,472 | 25 | 54 | 14 | 15 | 0,419 | 0,731 |
| 90,000 | 0,625 | 0,470 | 0,758 | 0,824 | 0,714 | 0,897 | 0,676 | 0,789 | 3,542 | 0,455 | 25 | 56 | 12 | 15 | 0,449 | 0,750 |
| 91,000 | 0,625 | 0,470 | 0,758 | 0,838 | 0,731 | 0,908 | 0,694 | 0,792 | 3,864 | 0,447 | 25 | 57 | 11 | 15 | 0,463 | 0,759 |
| **92,000** | **0,625** | **0,470** | **0,758** | **0,853** | **0,747** | **0,919** | **0,714** | **0,795** | **4,250** | **0,440** | **25** | **58** | **10** | 15 | **0,478** | **0,769** |
| 93,000 | 0,600 | 0,446 | 0,736 | 0,853 | 0,747 | 0,919 | 0,706 | 0,784 | 4,080 | 0,469 | 24 | 58 | 10 | 16 | 0,453 | 0,759 |
| 94,000 | 0,575 | 0,422 | 0,715 | 0,853 | 0,747 | 0,919 | 0,697 | 0,773 | 3,910 | 0,498 | 23 | 58 | 10 | 17 | 0,428 | 0,750 |
| 95,000 | 0,550 | 0,398 | 0,693 | 0,853 | 0,747 | 0,919 | 0,688 | 0,763 | 3,740 | 0,528 | 22 | 58 | 10 | 18 | 0,403 | 0,741 |
| 96,000 | 0,550 | 0,398 | 0,693 | 0,882 | 0,781 | 0,941 | 0,733 | 0,769 | 4,675 | 0,510 | 22 | 60 | 8 | 18 | 0,432 | 0,759 |
| 100,000 | 0,500 | 0,352 | 0,648 | 0,882 | 0,781 | 0,941 | 0,714 | 0,750 | 4,250 | 0,567 | 20 | 60 | 8 | 20 | 0,382 | 0,741 |
| 101,000 | 0,475 | 0,330 | 0,625 | 0,882 | 0,781 | 0,941 | 0,704 | 0,741 | 4,038 | 0,595 | 19 | 60 | 8 | 21 | 0,357 | 0,731 |
| 102,000 | 0,450 | 0,307 | 0,602 | 0,897 | 0,799 | 0,951 | 0,720 | 0,735 | 4,371 | 0,613 | 18 | 61 | 7 | 22 | 0,347 | 0,731 |
| 103,000 | 0,425 | 0,285 | 0,578 | 0,912 | 0,816 | 0,961 | 0,739 | 0,729 | 4,817 | 0,631 | 17 | 62 | 6 | 23 | 0,337 | 0,731 |
| 104,000 | 0,400 | 0,264 | 0,554 | 0,912 | 0,816 | 0,961 | 0,727 | 0,721 | 4,533 | 0,658 | 16 | 62 | 6 | 24 | 0,312 | 0,722 |
| 106,000 | 0,375 | 0,242 | 0,530 | 0,926 | 0,834 | 0,971 | 0,750 | 0,716 | 5,100 | 0,675 | 15 | 63 | 5 | 25 | 0,301 | 0,722 |
| 108,000 | 0,350 | 0,221 | 0,506 | 0,941 | 0,853 | 0,981 | 0,778 | 0,711 | 5,950 | 0,691 | 14 | 64 | 4 | 26 | 0,291 | 0,722 |

Abbreviations: LB=Lower Bound; UB=Upper Bound; TP=True Positive; TN=True Negative; FP=False Positive; FN=False Negative; J=Youden-Index; ACC=Accuracy; row with optimal statistical cut-off by Youden-Index is outlined bold

**S2 Table C. Excerpts of the ROC-curve coordinates: ÖMPSQ outcome of functional ability.**

| ÖMPSQ Score Cut-Offs  (n=122) | Sensitivity | LB (95%) | UB (95%) | Specificity | LB (95%) | UB (95%) | PPV | NPV | PLR | NLR | TP | TN | FP | FN | J | ACC |
| --- | --- | --- | --- | --- | --- | --- | --- | --- | --- | --- | --- | --- | --- | --- | --- | --- |
| 56,000 | 1,000 | 0,930 | 1,000 | 0,328 | 0,221 | 0,457 | 0,621 | 1,000 | 1,487 | 0,000 | 64 | 19 | 39 | 0 | 0,328 | 0,680 |
| 59,000 | 1,000 | 0,930 | 1,000 | 0,362 | 0,251 | 0,491 | 0,634 | 1,000 | 1,568 | 0,000 | 64 | 21 | 37 | 0 | 0,362 | 0,697 |
| 61,000 | 1,000 | 0,930 | 1,000 | 0,431 | 0,312 | 0,559 | 0,660 | 1,000 | 1,758 | 0,000 | 64 | 25 | 33 | 0 | 0,431 | 0,730 |
| 63,000 | 1,000 | 0,930 | 1,000 | 0,448 | 0,328 | 0,575 | 0,667 | 1,000 | 1,813 | 0,000 | 64 | 26 | 32 | 0 | 0,448 | 0,738 |
| 64,000 | 1,000 | 0,930 | 1,000 | 0,466 | 0,344 | 0,592 | 0,674 | 1,000 | 1,871 | 0,000 | 64 | 27 | 31 | 0 | 0,466 | 0,746 |
| 65,000 | 0,984 | 0,907 | 1,000 | 0,483 | 0,359 | 0,608 | 0,677 | 0,966 | 1,903 | 0,032 | 63 | 28 | 30 | 1 | 0,467 | 0,746 |
| 67,000 | 0,984 | 0,907 | 1,000 | 0,517 | 0,392 | 0,641 | 0,692 | 0,968 | 2,039 | 0,030 | 63 | 30 | 28 | 1 | 0,502 | 0,762 |
| 68,000 | 0,984 | 0,907 | 1,000 | 0,534 | 0,408 | 0,656 | 0,700 | 0,969 | 2,115 | 0,029 | 63 | 31 | 27 | 1 | 0,519 | 0,770 |
| 69,000 | 0,969 | 0,885 | 0,997 | 0,534 | 0,408 | 0,656 | 0,697 | 0,939 | 2,081 | 0,058 | 62 | 31 | 27 | 2 | 0,503 | 0,762 |
| 70,000 | 0,969 | 0,885 | 0,997 | 0,552 | 0,425 | 0,672 | 0,705 | 0,941 | 2,161 | 0,057 | 62 | 32 | 26 | 2 | 0,520 | 0,770 |
| **72,000** | **0,969** | **0,885** | **0,997** | **0,569** | **0,441** | **0,688** | **0,713** | **0,943** | **2,248** | **0,055** | **62** | **33** | **25** | **2** | **0,538** | **0,779** |
| 74,000 | 0,938 | 0,844 | 0,979 | 0,569 | 0,441 | 0,688 | 0,706 | 0,892 | 2,175 | 0,110 | 60 | 33 | 25 | 4 | 0,506 | 0,762 |
| 75,000 | 0,938 | 0,844 | 0,979 | 0,586 | 0,458 | 0,703 | 0,714 | 0,895 | 2,266 | 0,107 | 60 | 34 | 24 | 4 | 0,524 | 0,770 |
| 76,000 | 0,922 | 0,825 | 0,969 | 0,603 | 0,475 | 0,719 | 0,720 | 0,875 | 2,325 | 0,129 | 59 | 35 | 23 | 5 | 0,525 | 0,770 |
| 77,000 | 0,891 | 0,787 | 0,948 | 0,621 | 0,492 | 0,734 | 0,722 | 0,837 | 2,348 | 0,176 | 57 | 36 | 22 | 7 | 0,511 | 0,762 |
| 78,000 | 0,844 | 0,733 | 0,914 | 0,655 | 0,526 | 0,764 | 0,730 | 0,792 | 2,447 | 0,238 | 54 | 38 | 20 | 10 | 0,499 | 0,754 |
| 79,000 | 0,813 | 0,698 | 0,890 | 0,655 | 0,526 | 0,764 | 0,722 | 0,760 | 2,356 | 0,286 | 52 | 38 | 20 | 12 | 0,468 | 0,738 |
| 80,000 | 0,797 | 0,681 | 0,878 | 0,655 | 0,526 | 0,764 | 0,718 | 0,745 | 2,311 | 0,310 | 51 | 38 | 20 | 13 | 0,452 | 0,730 |
| 81,000 | 0,750 | 0,630 | 0,840 | 0,672 | 0,543 | 0,779 | 0,716 | 0,709 | 2,289 | 0,372 | 48 | 39 | 19 | 16 | 0,422 | 0,713 |
| 82,000 | 0,734 | 0,614 | 0,827 | 0,707 | 0,579 | 0,808 | 0,734 | 0,707 | 2,506 | 0,376 | 47 | 41 | 17 | 17 | 0,441 | 0,721 |
| 83,000 | 0,719 | 0,598 | 0,814 | 0,759 | 0,633 | 0,851 | 0,767 | 0,710 | 2,978 | 0,371 | 46 | 44 | 14 | 18 | 0,477 | 0,738 |
| 84,000 | 0,703 | 0,581 | 0,801 | 0,759 | 0,633 | 0,851 | 0,763 | 0,698 | 2,913 | 0,391 | 45 | 44 | 14 | 19 | 0,462 | 0,730 |
| 85,000 | 0,625 | 0,502 | 0,733 | 0,759 | 0,633 | 0,851 | 0,741 | 0,647 | 2,589 | 0,494 | 40 | 44 | 14 | 24 | 0,384 | 0,689 |
| 88,000 | 0,594 | 0,471 | 0,705 | 0,759 | 0,633 | 0,851 | 0,731 | 0,629 | 2,460 | 0,536 | 38 | 44 | 14 | 26 | 0,352 | 0,672 |
| 89,000 | 0,578 | 0,456 | 0,691 | 0,793 | 0,670 | 0,878 | 0,755 | 0,630 | 2,794 | 0,532 | 37 | 46 | 12 | 27 | 0,371 | 0,680 |
| 90,000 | 0,547 | 0,426 | 0,662 | 0,793 | 0,670 | 0,878 | 0,745 | 0,613 | 2,643 | 0,571 | 35 | 46 | 12 | 29 | 0,340 | 0,664 |
| 91,000 | 0,547 | 0,426 | 0,662 | 0,810 | 0,689 | 0,892 | 0,761 | 0,618 | 2,884 | 0,559 | 35 | 47 | 11 | 29 | 0,357 | 0,672 |
| 92,000 | 0,531 | 0,411 | 0,648 | 0,810 | 0,689 | 0,892 | 0,756 | 0,610 | 2,801 | 0,578 | 34 | 47 | 11 | 30 | 0,342 | 0,664 |
| 93,000 | 0,531 | 0,411 | 0,648 | 0,828 | 0,708 | 0,905 | 0,773 | 0,615 | 3,081 | 0,566 | 34 | 48 | 10 | 30 | 0,359 | 0,672 |
| 94,000 | 0,516 | 0,396 | 0,633 | 0,828 | 0,708 | 0,905 | 0,767 | 0,608 | 2,991 | 0,585 | 33 | 48 | 10 | 31 | 0,343 | 0,664 |
| 95,000 | 0,500 | 0,381 | 0,619 | 0,828 | 0,708 | 0,905 | 0,762 | 0,600 | 2,900 | 0,604 | 32 | 48 | 10 | 32 | 0,328 | 0,656 |
| 96,000 | 0,469 | 0,352 | 0,589 | 0,845 | 0,727 | 0,918 | 0,769 | 0,590 | 3,021 | 0,629 | 30 | 49 | 9 | 34 | 0,314 | 0,648 |
| 100,000 | 0,438 | 0,323 | 0,559 | 0,845 | 0,727 | 0,918 | 0,757 | 0,576 | 2,819 | 0,666 | 28 | 49 | 9 | 36 | 0,282 | 0,631 |
| 101,000 | 0,422 | 0,309 | 0,544 | 0,845 | 0,727 | 0,918 | 0,750 | 0,570 | 2,719 | 0,684 | 27 | 49 | 9 | 37 | 0,267 | 0,623 |
| 102,000 | 0,391 | 0,281 | 0,513 | 0,879 | 0,767 | 0,943 | 0,781 | 0,567 | 3,237 | 0,693 | 25 | 51 | 7 | 39 | 0,270 | 0,623 |
| 103,000 | 0,359 | 0,253 | 0,482 | 0,879 | 0,767 | 0,943 | 0,767 | 0,554 | 2,978 | 0,729 | 23 | 51 | 7 | 41 | 0,239 | 0,607 |
| 104,000 | 0,359 | 0,253 | 0,482 | 0,897 | 0,788 | 0,954 | 0,793 | 0,559 | 3,474 | 0,715 | 23 | 52 | 6 | 41 | 0,256 | 0,615 |
| 106,000 | 0,344 | 0,239 | 0,467 | 0,914 | 0,808 | 0,966 | 0,815 | 0,558 | 3,988 | 0,718 | 22 | 53 | 5 | 42 | 0,258 | 0,615 |
| 108,000 | 0,328 | 0,226 | 0,451 | 0,931 | 0,830 | 0,977 | 0,840 | 0,557 | 4,758 | 0,722 | 21 | 54 | 4 | 43 | 0,259 | 0,615 |

Abbreviations: LB=Lower Bound; UB=Upper Bound; TP=True Positive; TN=True Negative; FP=False Positive; FN=False Negative; J=Youden-Index; ACC=Accuracy; row with optimal statistical cut-off by Youden-Index is outlined bold

**S2 Table D. Excerpts from the ROC-curve coordinates: HKF-R 10 outcome of pain.**

| HKF-R 10 Score Cut-Offs  (n=128) | Sensitivity | LB (95%) | UB (95%) | Specificity | LB (95%) | UB (95%) | PPV | NPV | PLR | NLR | TP | TN | FP | FN | J | ACC |
| --- | --- | --- | --- | --- | --- | --- | --- | --- | --- | --- | --- | --- | --- | --- | --- | --- |
| 19,700 | 0,856 | 0,766 | 0,914 | 0,263 | 0,149 | 0,422 | 0,733 | 0,435 | 1,161 | 0,549 | 77 | 10 | 28 | 13 | 0,119 | 0,680 |
| 20,100 | 0,856 | 0,766 | 0,914 | 0,289 | 0,170 | 0,449 | 0,740 | 0,458 | 1,204 | 0,499 | 77 | 11 | 27 | 13 | 0,145 | 0,688 |
| 20,600 | 0,844 | 0,754 | 0,906 | 0,289 | 0,170 | 0,449 | 0,738 | 0,440 | 1,188 | 0,537 | 76 | 11 | 27 | 14 | 0,134 | 0,680 |
| 20,900 | 0,833 | 0,741 | 0,897 | 0,289 | 0,170 | 0,449 | 0,735 | 0,423 | 1,173 | 0,576 | 75 | 11 | 27 | 15 | 0,123 | 0,672 |
| 20,900 | 0,822 | 0,729 | 0,888 | 0,289 | 0,170 | 0,449 | 0,733 | 0,407 | 1,157 | 0,614 | 74 | 11 | 27 | 16 | 0,112 | 0,664 |
| 21,100 | 0,811 | 0,717 | 0,879 | 0,289 | 0,170 | 0,449 | 0,730 | 0,393 | 1,142 | 0,653 | 73 | 11 | 27 | 17 | 0,101 | 0,656 |
| 23,000 | 0,811 | 0,717 | 0,879 | 0,316 | 0,191 | 0,476 | 0,737 | 0,414 | 1,185 | 0,598 | 73 | 12 | 26 | 17 | 0,127 | 0,664 |
| 23,400 | 0,811 | 0,717 | 0,879 | 0,342 | 0,212 | 0,502 | 0,745 | 0,433 | 1,233 | 0,552 | 73 | 13 | 25 | 17 | 0,153 | 0,672 |
| 24,900 | 0,811 | 0,717 | 0,879 | 0,368 | 0,234 | 0,528 | 0,753 | 0,452 | 1,284 | 0,513 | 73 | 14 | 24 | 17 | 0,180 | 0,680 |
| 25,800 | 0,811 | 0,717 | 0,879 | 0,395 | 0,256 | 0,553 | 0,760 | 0,469 | 1,340 | 0,479 | 73 | 15 | 23 | 17 | 0,206 | 0,688 |
| 26,000 | 0,800 | 0,704 | 0,870 | 0,395 | 0,256 | 0,553 | 0,758 | 0,455 | 1,322 | 0,507 | 72 | 15 | 23 | 18 | 0,195 | 0,680 |
| 26,300 | 0,800 | 0,704 | 0,870 | 0,421 | 0,279 | 0,578 | 0,766 | 0,471 | 1,382 | 0,475 | 72 | 16 | 22 | 18 | 0,221 | 0,688 |
| 27,100 | 0,789 | 0,692 | 0,861 | 0,421 | 0,279 | 0,578 | 0,763 | 0,457 | 1,363 | 0,501 | 71 | 16 | 22 | 19 | 0,210 | 0,680 |
| 27,500 | 0,778 | 0,680 | 0,852 | 0,421 | 0,279 | 0,578 | 0,761 | 0,444 | 1,343 | 0,528 | 70 | 16 | 22 | 20 | 0,199 | 0,672 |
| 27,900 | 0,767 | 0,668 | 0,842 | 0,421 | 0,279 | 0,578 | 0,758 | 0,432 | 1,324 | 0,554 | 69 | 16 | 22 | 21 | 0,188 | 0,664 |
| 28,100 | 0,767 | 0,668 | 0,842 | 0,447 | 0,302 | 0,603 | 0,767 | 0,447 | 1,387 | 0,522 | 69 | 17 | 21 | 21 | 0,214 | 0,672 |
| 28,200 | 0,756 | 0,657 | 0,833 | 0,447 | 0,302 | 0,603 | 0,764 | 0,436 | 1,367 | 0,546 | 68 | 17 | 21 | 22 | 0,203 | 0,664 |
| 28,300 | 0,733 | 0,633 | 0,814 | 0,447 | 0,302 | 0,603 | 0,759 | 0,415 | 1,327 | 0,596 | 66 | 17 | 21 | 24 | 0,181 | 0,648 |
| 28,600 | 0,733 | 0,633 | 0,814 | 0,474 | 0,325 | 0,627 | 0,767 | 0,429 | 1,393 | 0,563 | 66 | 18 | 20 | 24 | 0,207 | 0,656 |
| 29,900 | 0,722 | 0,621 | 0,804 | 0,474 | 0,325 | 0,627 | 0,765 | 0,419 | 1,372 | 0,586 | 65 | 18 | 20 | 25 | 0,196 | 0,648 |
| 30,500 | 0,711 | 0,610 | 0,795 | 0,474 | 0,325 | 0,627 | 0,762 | 0,409 | 1,351 | 0,610 | 64 | 18 | 20 | 26 | 0,185 | 0,641 |
| 30,800 | 0,700 | 0,598 | 0,785 | 0,474 | 0,325 | 0,627 | 0,759 | 0,400 | 1,330 | 0,633 | 63 | 18 | 20 | 27 | 0,174 | 0,633 |
| 31,200 | 0,689 | 0,587 | 0,775 | 0,474 | 0,325 | 0,627 | 0,756 | 0,391 | 1,309 | 0,657 | 62 | 18 | 20 | 28 | 0,163 | 0,625 |
| 31,400 | 0,689 | 0,587 | 0,775 | 0,500 | 0,349 | 0,651 | 0,765 | 0,404 | 1,378 | 0,622 | 62 | 19 | 19 | 28 | 0,189 | 0,633 |
| 32,200 | 0,689 | 0,587 | 0,775 | 0,526 | 0,373 | 0,675 | 0,775 | 0,417 | 1,454 | 0,591 | 62 | 20 | 18 | 28 | 0,215 | 0,641 |
| 32,300 | 0,678 | 0,575 | 0,765 | 0,526 | 0,373 | 0,675 | 0,772 | 0,408 | 1,431 | 0,612 | 61 | 20 | 18 | 29 | 0,204 | 0,633 |
| 33,200 | 0,678 | 0,575 | 0,765 | 0,553 | 0,397 | 0,698 | 0,782 | 0,420 | 1,515 | 0,583 | 61 | 21 | 17 | 29 | 0,230 | 0,641 |
| 33,400 | 0,667 | 0,564 | 0,755 | 0,553 | 0,397 | 0,698 | 0,779 | 0,412 | 1,490 | 0,603 | 60 | 21 | 17 | 30 | 0,219 | 0,633 |
| 34,300 | 0,667 | 0,564 | 0,755 | 0,579 | 0,422 | 0,721 | 0,789 | 0,423 | 1,583 | 0,576 | 60 | 22 | 16 | 30 | 0,246 | 0,641 |
| 35,300 | 0,656 | 0,552 | 0,745 | 0,605 | 0,447 | 0,744 | 0,797 | 0,426 | 1,661 | 0,569 | 59 | 23 | 15 | 31 | 0,261 | 0,641 |
| 35,500 | 0,644 | 0,541 | 0,735 | 0,605 | 0,447 | 0,744 | 0,795 | 0,418 | 1,633 | 0,587 | 58 | 23 | 15 | 32 | 0,250 | 0,633 |
| 35,600 | 0,633 | 0,530 | 0,725 | 0,605 | 0,447 | 0,744 | 0,792 | 0,411 | 1,604 | 0,606 | 57 | 23 | 15 | 33 | 0,239 | 0,625 |
| 36,300 | 0,622 | 0,519 | 0,715 | 0,605 | 0,447 | 0,744 | 0,789 | 0,404 | 1,576 | 0,624 | 56 | 23 | 15 | 34 | 0,227 | 0,617 |
| 36,500 | 0,622 | 0,519 | 0,715 | 0,632 | 0,472 | 0,766 | 0,800 | 0,414 | 1,689 | 0,598 | 56 | 24 | 14 | 34 | 0,254 | 0,625 |
| 36,900 | 0,611 | 0,508 | 0,705 | 0,658 | 0,498 | 0,788 | 0,809 | 0,417 | 1,786 | 0,591 | 55 | 25 | 13 | 35 | 0,269 | 0,625 |
| 38,000 | 0,600 | 0,497 | 0,695 | 0,658 | 0,498 | 0,788 | 0,806 | 0,410 | 1,754 | 0,608 | 54 | 25 | 13 | 36 | 0,258 | 0,617 |
| 38,100 | 0,600 | 0,497 | 0,695 | 0,684 | 0,524 | 0,809 | 0,818 | 0,419 | 1,900 | 0,585 | 54 | 26 | 12 | 36 | 0,284 | 0,625 |
| 38,600 | 0,589 | 0,486 | 0,685 | 0,711 | 0,551 | 0,830 | 0,828 | 0,422 | 2,034 | 0,579 | 53 | 27 | 11 | 37 | 0,299 | 0,625 |
| 39,600 | 0,589 | 0,486 | 0,685 | 0,737 | 0,578 | 0,851 | 0,841 | 0,431 | 2,238 | 0,558 | 53 | 28 | 10 | 37 | 0,326 | 0,633 |
| 40,300 | 0,578 | 0,475 | 0,674 | 0,737 | 0,578 | 0,851 | 0,839 | 0,424 | 2,196 | 0,573 | 52 | 28 | 10 | 38 | 0,315 | 0,625 |
| 41,200 | 0,567 | 0,464 | 0,664 | 0,737 | 0,578 | 0,851 | 0,836 | 0,418 | 2,153 | 0,588 | 51 | 28 | 10 | 39 | 0,304 | 0,617 |
| 43,100 | 0,544 | 0,442 | 0,643 | 0,737 | 0,578 | 0,851 | 0,831 | 0,406 | 2,069 | 0,618 | 49 | 28 | 10 | 41 | 0,281 | 0,602 |
| 43,900 | 0,544 | 0,442 | 0,643 | 0,763 | 0,605 | 0,871 | 0,845 | 0,414 | 2,299 | 0,597 | 49 | 29 | 9 | 41 | 0,308 | 0,609 |
| 44,500 | 0,533 | 0,431 | 0,633 | 0,763 | 0,605 | 0,871 | 0,842 | 0,408 | 2,252 | 0,611 | 48 | 29 | 9 | 42 | 0,296 | 0,602 |
| 44,900 | 0,533 | 0,431 | 0,633 | 0,789 | 0,633 | 0,891 | 0,857 | 0,417 | 2,533 | 0,591 | 48 | 30 | 8 | 42 | 0,323 | 0,609 |
| 45,400 | 0,511 | 0,410 | 0,612 | 0,816 | 0,662 | 0,910 | 0,868 | 0,413 | 2,775 | 0,599 | 46 | 31 | 7 | 44 | 0,327 | 0,602 |
| 46,000 | 0,500 | 0,399 | 0,601 | 0,816 | 0,662 | 0,910 | 0,865 | 0,408 | 2,714 | 0,613 | 45 | 31 | 7 | 45 | 0,316 | 0,594 |
| **46,800** | **0,500** | **0,399** | **0,601** | **0,842** | **0,691** | **0,928** | **0,882** | **0,416** | **3,167** | **0,594** | **45** | **32** | **6** | **45** | **0,342** | **0,602** |
| 47,100 | 0,489 | 0,388 | 0,590 | 0,842 | 0,691 | 0,928 | 0,880 | 0,410 | 3,096 | 0,607 | 44 | 32 | 6 | 46 | 0,331 | 0,594 |
| 47,300 | 0,478 | 0,378 | 0,580 | 0,842 | 0,691 | 0,928 | 0,878 | 0,405 | 3,026 | 0,620 | 43 | 32 | 6 | 47 | 0,320 | 0,586 |
| 48,100 | 0,467 | 0,367 | 0,569 | 0,842 | 0,691 | 0,928 | 0,875 | 0,400 | 2,956 | 0,633 | 42 | 32 | 6 | 48 | 0,309 | 0,578 |

Abbreviations: LB=Lower Bound; UB=Upper Bound; TP=True Positive; TN=True Negative; FP=False Positive; FN=False Negative; J=Youden-Index; ACC=Accuracy; row with optimal statistical cut-off by Youden-Index is outlined bold
